# A case of Adrenocoricotrophic hormone -independent bilateral adrenocortical macronodular hyperplasia concomitant with primary aldosteronism

**DOI:** 10.1186/s12893-017-0293-z

**Published:** 2017-09-06

**Authors:** Mao Tokumoto, Naoyoshi Onoda, Yukie Tauchi, Shinichiro Kashiwagi, Satoru Noda, Norikazu Toi, Masahumi Kurajoh, Masahiko Ohsawa, Yuto Yamazaki, Hironobu Sasano, Kosei Hirakawa, Masaichi Ohira

**Affiliations:** 1 Department of Surgical Oncology, Osaka City University Graduate School of Medicine, 1-4-3 Asahi-machi, Abeno-ku, Osaka, 545-8585 Japan; 2 Department of Metabolism, Endocrinology and Molecular Medicine, Osaka City University Graduate School of Medicine, Osaka, Japan; 3 Department of Diagnostic Pathology, Osaka City University Graduate School of Medicine, Osaka, Japan; 4 Department of Anatomic Pathology, Tohoku University Graduate school of Medicine, Sendai, Japan

**Keywords:** ACTH – Independent bilateral adrenocortical macronodular hyperplasia, Primary aldosteronism, Laparoscopic unilateral adrenolectomy, Preclinical Cushing’s syndrome

## Abstract

**Background:**

Adrenocoricotrophic hormone (ACTH) – independent bilateral adrenocortical macronodular hyperplasia (AIMAH) is a rare cause of Cushing’s syndrome, and is characterized by bilateral adrenal hyperplasia. However, Primary aldosteronism (PA) is a relatively common adrenal disease.

**Case presentation:**

A 56-year-old man who has been treated hypertension and diabetes mellitus was detected low plasma potassium level with an elevated level of plasma aldosterone concentration and bilateral adrenal swelling. Endocrinological examinations showed autonomous secretion of cortisol and aldosterone, with suppression of plasma ACTH level and renin activity. A selective adrenal venous sampling demonstrated that left adrenal gland was responsible for aldosterone hypersecretion. He was diagnosed preclinical Cushing’s syndrome due to ACTH – independent bilateral adrenocortical macronodular hyperplasia (AIMAH) associated with aldosterone producing adenoma of the left adrenal gland. A laparoscopic left adrenalectomy was performed.

**Conclusion:**

The resected adrenal specimen histologically consisted with a diagnosis of AIMAH. Moreover, tiny cell clusters positive immunostaining for aldosterone synthase was revealed. This is a rare case of AIMAH accompanied by preclinical Cushing’s syndrome and primary aldosteronism.

## Background

Adrenocoricotrophic hormone (ACTH) – independent bilateral adrenocortical macronodular hyperplasia (AIMAH) is a disease which shows Cushing’s syndrome or preclinical Cushing’s syndrome due to autonomous cortisol secretion, and is characterized by bilateral adrenal hyperplasia [1]. AIMAH is a rare cause of Cushing’s syndrome, and it accounts for less than 1% of the Cushing’s syndrome. Recently, AIMAH has been found incidentally by abdominal imaging. Primary aldosteronism (PA), on the other hand, is a relatively common adrenal disease that causes secondary hypertension by excess aldosterone production either from the adreno-cortical adenoma or the hyperplasia. As far as we know, there were few reports of AIMAH associated with PA [2–4]. Herein, we report a rare case of AIMAH accompanied by preclinical Cushing’s syndrome and primary aldosteronism due to unilateral multiple adrenal micronodules (UMN), which localized by adrenal venous sampling and confirmed by immunohistological analysis of the resected adrenal apecimen.

## Case presentation

A 56-year-old man has been treated hypertension and diabetes mellitus for several years at a local physician. He had received annual medical check-up, and was detected to have low plasma potassium level with an elevated level of plasma aldosterone concentration (PAC)/ plasma renin activity (PRA) ratio of 940. Bilateral adrenal swelling was demonstrated by an abdominal computed tomography (CT). So, he was referred to our hospital for further examinations and treatment. He had no family history of endocrine diseases. He was 168 cm tall and weighed 72 kg (body mass index: 25.4 kg/m^2^). He did not show any physical signs suggesting of Cushing’s syndrome, such as central obesity, skin atrophy, buffalo hump, red striae of skin or moon face. His blood pressure was managed to 134/77 mmHg with anti-hypertensive drugs, including doxazosin (4 mg) and nicardipine (40 mg). Laboratory examination (Table 1) showed a low plasma potassium level, a normal LDL-cholesterol level, and a normal fasting glucose level. Serum levels of cortisol and renin activity were within the normal range. Plasma ACTH level was slightly low, whereas serum level of aldosterone was high. In urinary extraction, cortisol was slightly high, and aldosterone was high.

**Table 1:** Laboratory and endocrinological data

Complete blood count			
White blood cells	5500/ml		
Red blood cells	466 × 10^4^/ml		
Hemoglobin	13.6 g/dl		
Platelets	17.3 × 10^4^/ml		
Biochemistry			
Total protein	6.1 g/dl	Na	143 mEq/l
Albumin	3.9 g/dl	K	3.3 mEq/l
Asparate aminotransferase	14 IU/l	Cl	104 mEq/l
Alanine aminotransferase	9 IU/l	Total cholesterol	135 mg/dl
Blood urea nitrogen	13 mg/dl	Fasting blood glucose	76 mg/dl
Creatinine	0.74 mg/dl	Hemoglobin A1c	5.60%
Endocrinology data		Urinary excretion	
Adrenocorticotropin	5.3 pg/ml	Cortisol	33.6 mg/day
Serum cortisol	13.3 mg/dl	Aldosterone	29.9 μg/day
Plasma alldsterone concentration	423 pg/ml		
Plasma renin activity	0.3 ng/ml/hr		

His serum cortisol levels had no diurnal rhythm, and plasma ACTH level was suppressed throughout the day. Tests for endocrine functions showed unsuppressed cortisol levels by dexamethasone suppression test (DST), hypo-responses of ACTH and cortisol to corticotropin releasing hormone (CRH) stimulation, and hyper-responses of cortisol and aldosterone to ACTH stimulation (Table 2). These data were consistent with subclinical Cushing’s syndrome. On the other hand, stimulation with captopril and furosemide plus upright posture showed suppressed level of PRA (Table2).

**Table 2:** Endocrinological examinations

a) Diurnal rhythm					
Clock Time	8:00	16:00	22:00		
ACTH(pg/ml)	1.2	1.3	<1.0		
Cortisol(μg/dl)	4.6	5.9	5.3		
b) Dexamethasone suppression tests					
Dexamethasone	1 mg	8 mg			
ACTH (pg/ml)	<1.0	<1.0			
Cortisol (μg/dl)	6.6	4.6			
c) CRH stimulation test					
Time (min)	0	30	60	90	120
ACTH (pg/ml)	3.4	18.2	9.1	4.2	3.3
Cortisol (μg/dl)	8.7	20.0	18.1	13.7	11.2
d) ACTH stimulation test					
Time (min)	0	30	60		
Cortisol (μg/dl)	8.7	25.7	27.8		
PAC (pg/ml)	242	720	925		
e) Furosemide plus upright test					
Time (min)	0	60	120		
PRA (ng/ml/h)	0.2	0.4	0.4		
PAC (pg/ml)	192	566	787		
f) Captopri loaded test					
Time (min)	0	60	90		
PRA (ng/ml/h)	0.2	0.2	0.2		
PAC (pg/ml)	323	232	210		

**Fig. 1:**
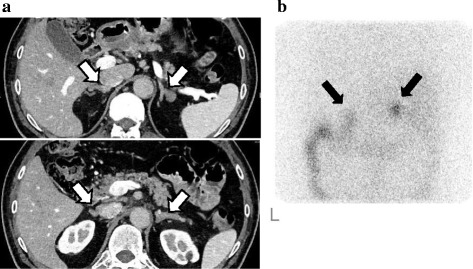
Clinical images. Abdominal CT scan shows nodular enlargement of bilateral adrenal glands (arrows) (**a**).^131^ I-Adosterol scintigraphy shows increased uptake of radioactivity to bilateral adrenal glands (**b**)

These data were consistent with PA. Abdominal CT revealed bilateral nodular masses (Fig. 1a), and ^131^I–Adsterol scintigraphy showed increased uptake of radioactivity at bilateral adrenal glands (Fig. 1b).

**Fig. 2:**
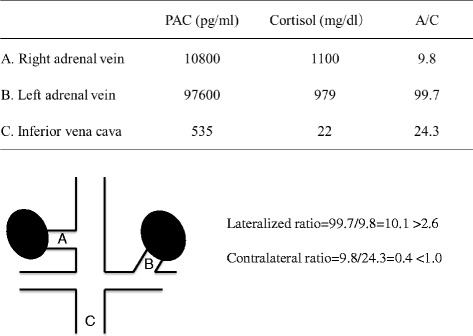
Selevtive adrenal venous sampling. The result of selective adrenal venous sampling indicates aldosterone hypersecretion from left adrenal gland. PAC: Plasma aldosterone concentration, A/C: PAC/Cortisol ratio

We performed a selective adrenal venous sampling (AVS) to confirm the lateralization of the lesion of excess aldosterone secretion. After ACTH stimulation, the PAC in the left adrenal vein was 97,600 pg/ml (range of diagnosis for laterality; greater than 14,000 pg/ml) the lateralized ratio of the left side to right side was 10.1 (>2.6), and the contralateral ratio was 0.4 (<1.0) (Fig. 2). These results indicated that left adrenal gland was responsible for aldosterone hypersecretion [5]. A laparoscopic left adrenalectomy was conducted in accordance to the diagnosis of AIMAH associated with aldosterone producing adenoma of the left adrenal gland. He was doing well after surgery, and left the hospital for six days. His blood pressure was 113/71 mmHg with reduced dose of doxazosin (2 mg) and nicardipine (40 mg). Five months after the surgery, serum cortisol, aldosterone, and plasma ACTH concentration were found to be within the normal range, 10.7 mg/dl, 101 pg/ml, and 11.2 pg/ml, respectively. No enlargement of the contralateral right adrenal gland was demonstrated.

**Fig. 3:**
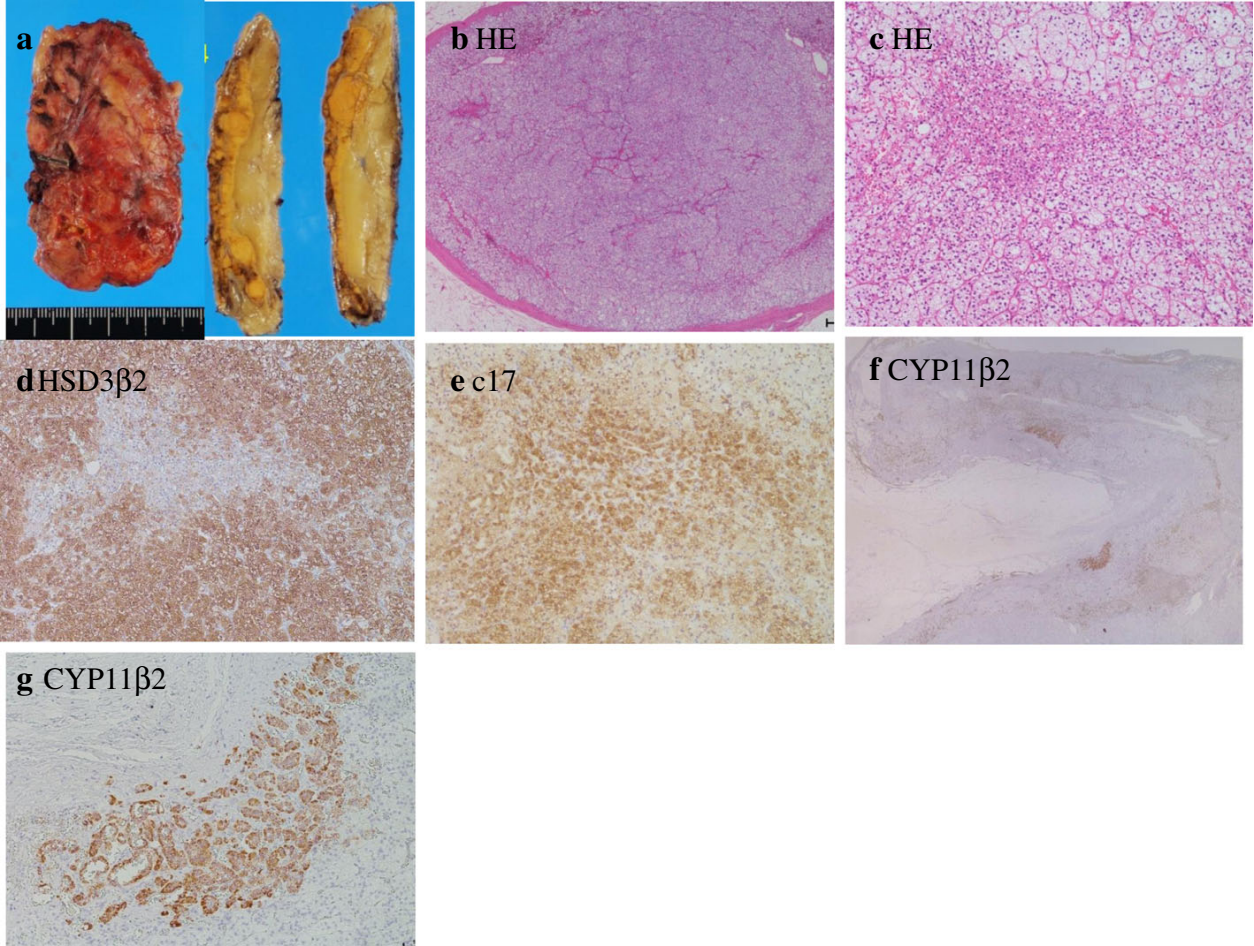
Macroscopic image and histological examination of the resected left adrenal specimen. Multiple macro-nodular lesions were found in the surgical specimen (**a**). Multiple capsulated nodules that were composed of large clear cells and small compact cells were identified by macroscopic examination. (**b**: magnification ×20, **c**: magnification ×100) Immunohistochemical staining showed large clear cells were positive for HSD3β2 (**d**: magnification ×100), and small compact cells were positive for CYP17 (**e**: magnification ×100). In the non-nodular adrenal cortex of zona glomerulosa, small cell clusters with positive immunostaining for CYP11β2 were identified, but not within the nodules. (**f**: magnification ×10, **g**: magnification ×100)

Macroscopically, the left adrenal gland was 3.8 × 6.7 cm in size, 30 g in weight, and comprised various sizes of yellow nodules (Fig. 3a). Histological findings revealed multiple capsulated macro-nodules that were composed of large clear cells stained with 3β-hydroxysteroid dehydrogenase (HSD3β2; 1:3000 dilution; M. Doi et al. J Clin Endocrinol Metab, 2014, 99(2), Kyoto, Japan) and small compact cells stained with 17β-hydroxylase (CYP17; 1:500 dilution; BEX, Tokyo, Japan), consisted with a diagnosis of AIMAH (Fig. 3b-e). Among non-nodular adrenal cortex, zona glomerulosa cell clusters, positive immunostaining for aldosterone synthase (CYP11β2; 1:500 dilution; C. Gomez-Sanchez et al. Mol Cell Endocrinol. 2014, 383(0), University of Missisippi Medical Center, Birmingham, AL, USA), were identified by the immunostaining (Fig. 3f-g).

## Discussion and conclusion

AIMAH was firstly reported in 1964 by Kirschner et al. as a bilateral adrenal hyperplastic disease which caused Cushing’s syndrome [6]. It was reported to have an even distribution between gender and a peak at the fifth and sixth decades [1].

In this case, low level of plasma ACTH concentration, normal plasma and urinary cortisol level, lack of diurnal rhythm, hypo-responses of ACTH and cortisol to CRH stimulation, and hyper-responses of cortisol and aldosterone to ACTH stimulation are consistent with a diagnosis of preclinical Cushing’s syndrome, and bilateral adrenal enlargement by abdominal CT scan and histological features are also consistent with a diagnosis of AIMAH. Namely, this patient showed symptoms of preclinical Cushing’s syndrome because of AIMAH. Moreover, poor PRA response to stimulation with captopril and furosemide plus upright posture and the results of AVS are compatible with PA by aldosterone hypersecretion from left adrenal gland. Therefore, we diagnosed the patients to have AIMAH accompanied with PA, even is a rare combination. As mentioned above, immunohistochemical staining pattern in macro-nodular lesion showed cortisol production consistent with AIMAH. It has been reported that the cortisol secretion capacity of the hyperplastic cells in AIMAH are not aggressive, and the plasma cortisol level significantly correlates with the volume of adrenal gland [7]. Because the adrenal glands in this patient were relatively small, remarkable elevation of serum cortisol level was not found.

Cortical adenoma which is the most common cause of PA was not found in the resected specimen, whereas CYP11β2 positive tiny cell clusters were identified among the non-nodular area of the adrenal cortex, but not within the nodules. These findings indicated that hypersecretion of aldosterone in this case was caused by UMN. In fact, this patient could decrease anti-hypertensive drugs. As well as his serum potassium level became normalized after left adrenalectomy.

Conventionally, bilateral adrenalectomy was the treatment of choice for patients with AIMAH, and life-time steroid replacement therapy is required afterwards. On the contrary, recent reports have shown that unilateral adrenalectomy of predominant side successfully improves hormonal status and clinical symptoms of the patient with AIMAH [2, 8]. In this case, we conducted left adrenalectomy because AVS showed the result of aldosterone hypersecretion from the left adrenal gland. His postoperative course has been passed without any problems. The limit of unilateral adrenalectomy is the possibility of recurrent disease due to the growth of the residual adrenal gland. In that case, contralateral adrenalectomy may have to be performed, followed by life-time steroid replacement therapy. Lately, it has been revealed that cortisol hyper secretion of AIMAH was caused by various ectopic receptors, such as gastric inhibitory polypeptide (GIP) receptor [9], vasopressin receptor [10], serotonin receptor,[11] luteinizing hormone/human chronic gonadotropin (LH/hCG) [12], and β-adrenergic receptor,[13] present in adrenocortical cells. The effectiveness of antagonist to these receptors has been reported [10, 12, 13].

In summary, we reported a rare case of AIMAH accompanied by PA due to UMN which is confirmed by a detailed histopathological examination. When the molecular physiology of AIMAH is clarified, medication with antagonist to specific receptor may become a possible alternative treatment to an invasive bilateral adrenalectomy, and this may avoid the life-time steroid replacement therapy. Further investigation and careful observation are needed to reveal the etiology of AIMAH and its long-term prognosis.

## Abbreviations

ACTH: Adrenocoricotrophic hormone
AIMAH: Adrenocoricotrophic hormone – independent bilateral adrenocortical macronodular hyperplasia
AVS: adrenal venous sampling
CRH: corticotropin releasing hormone
CT: computed tomography
CYP11β2: aldosterone synthase
CYP17: 17α-hydroxylase
DST: dexamethasone suppression test
GIP: gastric inhibitory polypeptide
HSD3β2: 3β-hydroxysteroid dehydrogenase
LH/hCG: luteinizing hormone/human chronic gonadotropin
PA: Primary aldosteronism
PAC: plasma aldosterone concentration
PRA: plasma renin activity
UMN: unilateral multiple adrenal micronodules
